# Evaluation of a Multimodal Anesthetic Protocol for Immobilization in Black Vultures (*Coragyps atratus*) and Turkey Vultures (*Cathartes aura*)

**DOI:** 10.3390/vetsci12111091

**Published:** 2025-11-16

**Authors:** Alejandro Vargas Araya, Jeff C. Ko, Tomohito Inoue, Shane Guenin, Tyler C. Hunt, Patrice E. Baumhardt, Esteban Fernández-Juricic

**Affiliations:** 1 Department of Veterinary Clinical Sciences, College of Veterinary Medicine, Purdue University, West Lafayette, IN 47907, USA; avargasa@purdue.edu (A.V.A.); tinoue@purdue.edu (T.I.); 2 Department of Biological Sciences, College of Sciences, Purdue University, West Lafayette, IN 47907, USA; guenins@purdue.edu (S.G.); tchunt@purdue.edu (T.C.H.); pbaumhar@purdue.edu (P.E.B.); efernan@purdue.edu (E.F.-J.)

**Keywords:** anesthesia, Black Vulture, Turkey Vulture, cardiorespiratory monitoring, electroretinography, isoflurane, ketamine, midazolam, dexmedetomidine, butorphanol, pressure-controlled ventilation

## Abstract

Anesthetic protocols for non-invasive immobilization of Black Vultures (*Coragyps atratus*) and Turkey Vultures (*Cathartes aura*) for procedures lasting up to two hours are lacking. This study directly evaluated the safety and efficacy of a multimodal anesthetic protocol in 11 Black Vultures and 4 Turkey Vultures undergoing electroretinography (ERG). Vultures were anesthetized with intramuscular dexmedetomidine (5 μg/kg), midazolam (0.2 mg/kg), butorphanol (0.2 mg/kg), and ketamine (5 mg/kg) (DMBK), followed by isoflurane induction and maintenance. All vultures were mechanically ventilated to maintain consistent end-tidal CO_2_. Monitored parameters included sedation and recovery quality, heart and respiratory rates, hemoglobin oxygen saturation, non-invasive blood pressure, body temperature, and end-tidal concentrations of CO_2_, isoflurane, and oxygen. All vultures achieved profound sedation with smooth induction and a median isoflurane maintenance concentration of 1.4% for approximately two hours. Recovery was rapid and uneventful. Heart rates ranged from 60 to 119 beats/min. Mean arterial blood pressure averaged 149 mmHg in Black Vultures and 158 mmHg in Turkey Vultures, with Turkey Vultures showing significantly higher diastolic pressure. A second-degree heart block was detected in one Black Vulture but required no treatment. All ERG procedures were completed successfully. The DMBK protocol provided profound sedation in both species, maintained key cardiorespiratory parameters, including heart rate and arterial blood pressure, within a clinically acceptable range throughout isoflurane maintenance, and enabled uneventful rapid recovery. These findings support DMBK with the cardiorespiratory monitoring system as a safe and effective regimen for anesthetizing vultures and likely other similarly sized raptors requiring non-invasive immobilization.

## Simple Summary

Black Vultures (*Coragyps atratus*) and Turkey Vultures (*Cathartes aura*) are obligate scavengers that soar at high altitudes. This behavior, unfortunately, places them at risk of colliding with aircraft. To better understand their vision and help mitigate these collisions, scientists use electroretinography (ERG) to study how the birds’ eyes respond to various light stimuli. However, this research requires the vultures to be safely anesthetized. Currently, there is a lack of established anesthesia and monitoring protocols for immobilizing these specific birds. This study evaluated an anesthetic combination and associated monitoring techniques in eleven adult Black Vultures and four adult Turkey Vultures. Following a 12–16 h fast, the vultures were premedicated with intramuscular dexmedetomidine, midazolam, butorphanol, and ketamine, and subsequently induced and maintained with isoflurane. All vultures were intubated and mechanically ventilated, and their electrocardiogram, arterial hemoglobin oxygen saturation, end-tidal CO_2_, isoflurane concentrations, non-invasive blood pressure, and body temperature were continuously monitored throughout the procedure. The anesthetic combination produced profound sedation and facilitated smooth induction. The anesthesia also effectively maintained a sufficient plane to suppress eye reflexes and ensure immobilization, both critical for uninterrupted ERG recording, while allowing for rapid, uneventful recoveries. Physiological responses were broadly similar between the two vulture species. Key physiological values, such as heart rate and arterial blood pressure, remained stable throughout the procedure, supporting the safety and clinical suitability of the protocol for ERG-based investigations. This protocol provided safe anesthesia and reliable monitoring and may apply to other bird species of similar size undergoing comparable procedures.

## 1. Introduction

The Black Vulture (*Coragyps atratus*) and Turkey Vulture (*Cathartes aura*) play a critical ecological role as obligate scavengers [1]. These birds consume carcasses. In doing so, they perform a vital ecological function by removing decomposing organic matter. This scavenging behavior helps prevent the accumulation of pathogens in the environment. As a result, they reduce disease transmission risks for both wildlife and humans [2,3]. Although these species often share the same habitats, their foraging strategies differ significantly. Anatomical and behavioral studies suggest that Turkey Vultures rely primarily on a well-developed sense of smell to locate carrion [4,5]. In contrast, Black Vultures depend more on sharp vision and social cues, often following other Black Vultures or Turkey Vultures to a carcass [6,7]. However, Turkey Vultures also possess acute vision comparable to that of Black Vultures [8], and Black Vultures can detect carrion by smell at distances up to 50 m [9].

Building on their distinct sensory strategies, it is paradoxical that the same behavioral and physiological traits that help vultures thrive also contribute to conflict in human-modified environments. Black and Turkey Vultures are among the few vulture species whose populations are currently increasing worldwide [2]. With minimal predation pressure, vultures exhibit limited anti-predator behaviors, typically restricted to death-feigning (thanatosis), in which the bird becomes motionless to mimic death, and chemically deterrent regurgitation, releasing foul-smelling gastric contents to repel threats [10,11,12,13]. These traits reflect their ecological niche and have likely contributed to their survival and increasing population numbers in human-modified environments. As their numbers grow, their large body size, flocking behavior, and soaring flight patterns, often concentrated near landfills, roadways, and open fields, heighten the risk of aircraft collisions [14,15,16]. Such events often result in serious injuries, underscoring the need for both veterinary intervention and preventative research. One promising approach involves studying their visual systems through electroretinography (ERG), which provides insight into sensory processing [17]. Because ERG requires prolonged immobilization, developing a safe and reliable anesthetic protocol is essential for advancing both clinical care and research.

The avian brain has followed a distinct evolutionary trajectory, diverging from the mammalian lineage for over 300 million years. Despite this divergence, birds retain a complex neural architecture capable of supporting consciousness, the principal target of general anesthetic drugs [18,19,20]. Consciousness, in the context of anesthesia, refers to the brain’s capacity to integrate and respond to sensory input [19,20]. This includes proprioception, muscle tone, and protective reflexes against noxious stimuli, all of which reflect coordinated activity across multiple neurochemical systems [19,20]. These functions are maintained by foundational neurotransmitter pathways, GABAergic (gamma-aminobutyric acid), adrenergic, opioidergic, and glutamatergic systems, that are conserved across vertebrate taxa [19,20,21,22,23,24,25]. Anesthetic agents act on these systems to disrupt consciousness and produce the desired clinical endpoints: unconsciousness, muscle relaxation, antinociception, and amnesia [19,20]. Because each anesthetic class targets a specific neurotransmitter pathway, a multimodal approach, combining agents from different pharmacologic classes, can create synergistic effects that more reliably achieve these endpoints.

Multimodal anesthesia thus offers a strategic advantage: it allows for lower doses of individual drugs, reducing side effects while enhancing physiological stability [20].

In contrast, mono-modal techniques, often relying on high concentrations of inhalants, can lead to pronounced cardiorespiratory depression, including bradycardia, hypotension, and respiratory compromise [18]. Attempts to mitigate these effects by lowering inhalant doses may induce significant oscillations in anesthetic depth, resulting in unstable anesthetic planes, characterized by fluctuating cardiovascular depression [18]. This instability requires constant vigilance, close monitoring, and prompt intervention.

A review of the veterinary literature reveals a notable lack of information on effective anesthetic protocols for Black Vultures, Turkey Vultures, and related species [18]. Given their unique physiology and the evolutionary conservation of key neurotransmitter systems, it is essential to evaluate anesthetic strategies that engage these pathways in a balanced, multimodal fashion. Doing so will not only improve clinical outcomes but also support ethical research practices by ensuring predictable and safe anesthetic responses in New World vultures.

The primary objective of this study was to evaluate the efficacy and safety of a specific multimodal anesthetic protocol in adult Black Vultures (*Coragyps atratus*) and Turkey Vultures (*Cathartes aura*), and to characterize its associated cardiorespiratory effects. The protocol involved injectable premedication with dexmedetomidine, midazolam, butorphanol, and ketamine (DMBK), followed by induction and maintenance using isoflurane.

To support this objective, the study pursued several focused aims centered on protocol assessment and species-specific characterization. These included developing a quantifiable scoring system to objectively evaluate sedation quality and recovery, monitoring key cardiorespiratory parameters including heart rate (HR), respiratory rate (RR), blood pressure (BP), and oxygen saturation (SpO_2_) throughout the anesthetic period, determining the isoflurane concentration required to maintain a stable plane of immobilization, and comparing physiological responses and anesthetic requirements between Black and Turkey Vultures to identify clinically relevant, species-specific considerations.

## 2. Materials and Methods

### 2.1. Animals

This prospective experimental study utilized eleven adult Black Vultures (*Coragyps atratus*) and four adult Turkey Vultures (*Cathartes aura*) with an approximate body weight of 2 kg (see Table 1).

The vultures used in this study were humanely captured in Indiana and provided by the United States Department of Agriculture, Animal and Plant Health Inspection Service, Wildlife Services, in accordance with the Migratory Bird Treaty Act. For five months prior to the study, the vultures were housed at the Purdue University Wildlife Area, a 159-acre property in Tippecanoe County, Indiana. During this acclimation period, they were fed a diet of whole fetal pig carcasses, supplemented by beef and pork trimmings from a local butcher. All vultures were deemed healthy based on the observation of normal behaviors, eating, and activity, and were classified as having an American Society of Anesthesiologists (ASA) physical status of I. Food, but not water, was withheld for 12–16 h prior to each procedure. This fasting interval was selected based on a proposed protocol aimed at minimizing the risk of regurgitation and aspiration by reducing crop content as much as possible. Given the limited species-specific data available, the study also assessed whether this fasting duration offered any clinical benefits or drawbacks, with the goal of refining future anesthetic recommendations for these raptors.

### 2.2. Ethical Statement

Vultures were captured in Indiana by the United States Department of Agriculture—Animal and Plant Health Inspection Service Wildlife Services under permit #MB21788A. Vultures were legally possessed and housed under the Indiana Department of Natural Resources Scientific Collections Permit #24123. The Purdue University Animal Care and Use Committee approved all animal use procedures (Protocol #1805001745).

### 2.3. Anesthetic and Monitoring Protocol

Prior to sedation, each vulture was weighed to ensure accurate drug dosing. A brief physical examination was conducted under gentle restraint, and baseline heart and respiratory rates were recorded then. To ensure the safety of both the vulture and the handler, physical restraint was performed using protective leather gloves and a coordinated technique. One hand secured the vulture’s legs to prevent scratching, while the other controlled the head and neck to prevent biting. Meanwhile, the handler’s arms held the bird’s body and wings firmly against their own torso to prevent wing flapping or extension. Any defensive behaviors, such as defecation or regurgitation, were noted throughout the handling process. In cases of regurgitation, the consistency of the vomitus was assessed and documented as either fluid or containing solid material, providing insight into the effectiveness of the pre-anesthetic fast.

#### 2.3.1. Sedation and Induction

Once securely restrained, each vulture was administered a single intramuscular (IM) injection using a 22-gauge, 1-inch (2.54 cm) needle into the left pectoral muscle containing dexmedetomidine (5 µg/kg; Dexased, Cronus Pharma LLC, East Brunswick, NJ, USA), midazolam (0.2 mg/kg; Nephron Pharmaceuticals Corporation, West Columbia, SC, USA), butorphanol (0.2 mg/kg; Dechra Veterinary Products, Overland Park, KS, USA), and ketamine (5 mg/kg; Nephron Pharmaceuticals Corporation, West Columbia, SC, USA). Following administration, birds were placed in large, quiet crates for a 15 min undisturbed sedative period, during which the onset and progression of sedation were closely observed. At the conclusion of this period, two parameters were formally assigned using standardized 5-point scoring systems: the Sedative Transition Quality Score (Supplementary Table S1) and the Final Sedative Degree Score (Supplementary Table S2). These scores ensured consistent and objective evaluation across individuals. After scoring, each vulture was wrapped in a towel to facilitate safe handling and transferred to an adjacent procedure room for further monitoring and instrumentation.

Induction was performed using an anesthetic machine (Mindray North America, Mahwah, NJ, USA), which delivered 6% isoflurane in 100% oxygen at a flow rate of 4 L/min via a face mask connected to a non-rebreathing circuit (Bain Circuit, Vetamac Inc., Rossville, IN, USA). Adequate anesthetic depth for intubation was confirmed by profound muscle relaxation, ease of beak opening and mandibular manipulation, and absence of the toe-pinch reflex. Orotracheal intubation was performed using a cuffed polyvinyl chloride endotracheal tube, with the cuff intentionally left deflated. The ease and quality of isoflurane face mask induction and subsequent intubation were evaluated using a standardized 5-point scoring system, as detailed in Supplementary Table S3, and the duration of induction was recorded to assess consistency and efficiency across individuals. The endotracheal tube size was documented for each bird to facilitate species-level comparisons.

#### 2.3.2. Anesthetic Maintenance and the Electroretinogram Procedure

Following intubation, vultures were connected to a semi-closed circuit on a Mindray Veta 5 anesthetic machine (Mindray North America, Mahwah, NJ, USA). Mechanical ventilation was immediately initiated using the integrated Mindray Veta 5 Smart Ventilator, set to a pressure-controlled mode. The peak inspiratory pressure (PIP) was adjusted between 5 and 8 cmH_2_O, and the RR was set to 8–12 breaths per minute to maintain end-tidal carbon dioxide (EtCO_2_) between 35 and 45 mmHg. Anesthesia was maintained with isoflurane in 1 L/min of 100% oxygen, with the vaporizer setting titrated to achieve a stable end-tidal concentration (Et-Iso) between 1.0 and 2.0%, adjusted based on the vulture’s response to procedural stimulation.

For ERG recording, each vulture was positioned in sternal recumbency, with complete immobilization maintained throughout the procedure. To prepare the eye, the inferior eyelid was gently retracted using medical tape (3M™ Durapore™ Medical Tape; Solventum Corporation, St. Paul, MN, USA) to prevent corneal obstruction. A contact electrode was then carefully placed on the corneal surface. If corneal reflexes were elicited, particularly in the medial canthus region, the Et-Iso concentration was increased to suppress this response. Anesthetic depth was adjusted to reliably inhibit nystagmus, corneal, and palpebral reflexes prior to initiating ERG trials. To eliminate ambient light and ensure consistent dark adaptation, the vulture was enclosed within a light-proof wooden box. An integrated camera system enabled remote observation during the procedure. Detailed methodology and ERG results are presented in a separate study.

#### 2.3.3. Physiological Monitoring

**Figure 1:**
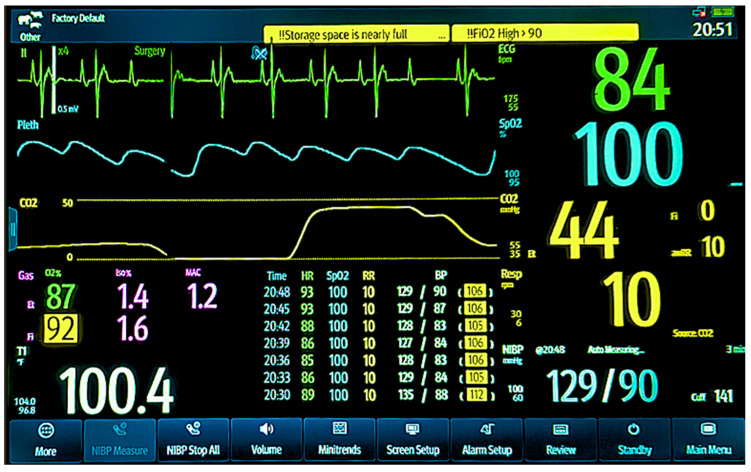
Multiparameter anesthetic monitoring display for a vulture. The lead II electrocardiogram (ECG, green tracing) shows a heart rate of 84 bpm with a second-degree atrioventricular (AV) block, Type I (Wenckebach), characterized by progressive P-R interval prolongation until a dropped beat. The pulse oximetry plethysmograph (blue tracing) confirms a pulse deficit consistent with the AV block and shows a hemoglobin oxygen saturation (SpO_2_) of 100%. Ventilatory parameters include an end-tidal CO_2_ (EtCO_2_, yellow tracing) of 44 mmHg, an inspiratory CO_2_ of 0, and a respiratory rate of 10 breaths/min. Anesthetic gas concentrations are 1.6% inspired (Fi-Iso) and 1.4% expired (Et-Iso) isoflurane, with 92% inspired (FiO_2_) and 87% expired (EtO_2_) oxygen. The patient’s body temperature is 100.4 °F. The most recent non-invasive blood pressure (NIBP) measurement was 129/90 mmHg, with a mean arterial pressure of 106 mmHg. A series of historical cardiorespiratory records for this vulture is visible in the lower middle portion of the screen, spanning from 20:30 to 20:48. These records demonstrate a consistent trend in heart rate (HR), SpO_2_, respiratory rate (RR), and blood pressure during isoflurane maintenance for the ERG procedure.

To ensure stable and continuous monitoring while each vulture remained physically isolated within a light-proof wooden box, cardiorespiratory parameters were remotely tracked using a Mindray ePM-12M Vet multiparameter monitor (Figure 1; Mindray North America, Mahwah, NJ, USA). HR and cardiac rhythm were monitored via a Lead II electrocardiogram (ECG) using non-traumatic alligator clips placed on the right wing (Right arm-RA), left wing (Left arm-LA), and left toe (Left leg-LL). Hemoglobin oxygen saturation (SpO_2_) was measured with a pulse oximeter probe positioned on the toe web. Indirect oscillometric BP, including systolic (SBP), diastolic (DBP), and mean arterial pressure (MBP), was recorded using a 3.1–5.7 cm cuff (CMA01 vet; Mindray Animal Care, Mindray North America, Mahwah, NJ, USA) placed just above the tibiotarsus joint, with cuff width selected to approximate 40% of limb circumference for optimal accuracy.

RR was regulated by a mechanical ventilator, while EtCO_2_, inspired (FiO_2_) and expired (EtO_2_) oxygen fractions, and Et-Iso concentrations were monitored via side-stream capnography using a neonatal setting with a 50 mL/min aspiration rate. Core body temperature was continuously measured using an esophageal probe and maintained between 100 °F and 106 °F through a combination of adjusting the room temperature, using towel insulation, and placing a water-heated blanket beneath the vulture. If signs of light anesthesia were observed, such as spontaneous movement, eye blinking, nystagmus, or changes in HR or BP, the vaporizer setting was promptly increased by 0.5–1.0% to restore and maintain an appropriate anesthetic depth once the vulture stabilized.

#### 2.3.4. Recovery

At the conclusion of the procedure, isoflurane administration was discontinued, and the vulture was disconnected from the mechanical ventilator. The bird was then transitioned to a Bain circuit delivering 100% oxygen, with manual ventilation provided until spontaneous breathing resumed consistently. Once protective airway reflexes returned, indicated by a swallow or gag response against the endotracheal tube, the vulture was extubated, and all monitoring equipment was removed. Following extubation, the bird was placed in sternal recumbency within a padded recovery crate to ensure comfort and stability. Recovery quality was formally assessed using a standardized scoring system, as outlined in Supplementary Table S4.

### 2.4. Statistical Analysis

All statistical analyses, with the exception of ordinal data (see below), were conducted using R version 4.5.1 (R Core Team. 2025, R: A language and environment for statistical computing, R Foundation for Statistical Computing, Vienna, Austria). Comparisons between the two vulture species for single-point variables, such as body weight, endotracheal tube size, and total anesthetic/ERG durations, were performed using two-sample *t*-tests. Ordinal data, specifically the induction and recovery scores, were compared using the Mann–Whitney U test via GraphPad Prism version 9.5.1 (GraphPad Software, San Diego, CA, USA).

Continuously monitored cardiorespiratory parameters (e.g., HR, BP, temperature, SpO_2_, EtCO_2_) were analyzed over time using general linear mixed models. These models included time and species, and their interaction (time × species) as fixed effects. For HR and RR models, time was included as a categorical factor to assess the comparison between baseline and time intervals for these factors. For models on all other parameters (BP, temperature, SpO_2_, EtCO_2_), time was included as a continuous factor to assess the rate of change over time (i.e., slopes) in the dependent variables. A random effect for individual vulture ID was included in all models to account for repeated measures from the same animal. Results from these general linear mixed models are presented as Estimated Marginal Means (EMM) ± Standard Error (SE). To visualize the trends identified by these models, key parameters (e.g., Heart Rate) were also plotted over time. Other descriptive data are presented as mean ± standard deviation (SD) or median and range [minimum–maximum], as appropriate for the data’s distribution.

## 3. Results

During initial pre-sedation handling, individual vultures exhibited a range of alert behaviors, spanning from overt defensiveness to active aggression. Common responses included biting at the handler’s leather gloves, vigorous wing flapping, forceful leg kicking, pronounced postural tension, and low-pitched cooing vocalizations. These behaviors reflected heightened arousal and stress induced by physical restraint, indicating a strong aversive response to handling.

Five of the fifteen vultures (33%) displayed liquid regurgitation and/or defecation responses. These behaviors ceased upon the onset of sedation, frequently occurring within three minutes following IM injection. All vultures became profoundly sedated by the end of the 15 min period, with a uniformly smooth transition and final sedation scores of 4 (Good) or 5 (Excellent) based on a standardized scoring system. This level of sedation allowed for calm and cooperative handling during transport to the procedure room. No discernible differences were noted between Black Vultures and Turkey Vultures in their awake behavior, response to sedation, or baseline HR.

Although sedation facilitated easy beak manipulation and a clear view of the glottis, initial intubation attempts were unsuccessful in all vultures due to a strong swallowing reflex triggered by the endotracheal tube. To proceed, anesthesia was induced via face mask with isoflurane, resulting in a rapid onset (1–2 min), successful intubation, and a smooth transition to maintenance anesthesia.

The mean body weights for Black and Turkey Vultures were similar, and no statistically significant differences were found between the species for induction score, endotracheal tube size, total anesthesia time, ERG recording time, or recovery score (Table 1). The median Et-Iso required for maintenance during the ERG procedures was 1.4% across the study.

**Figure 2:**
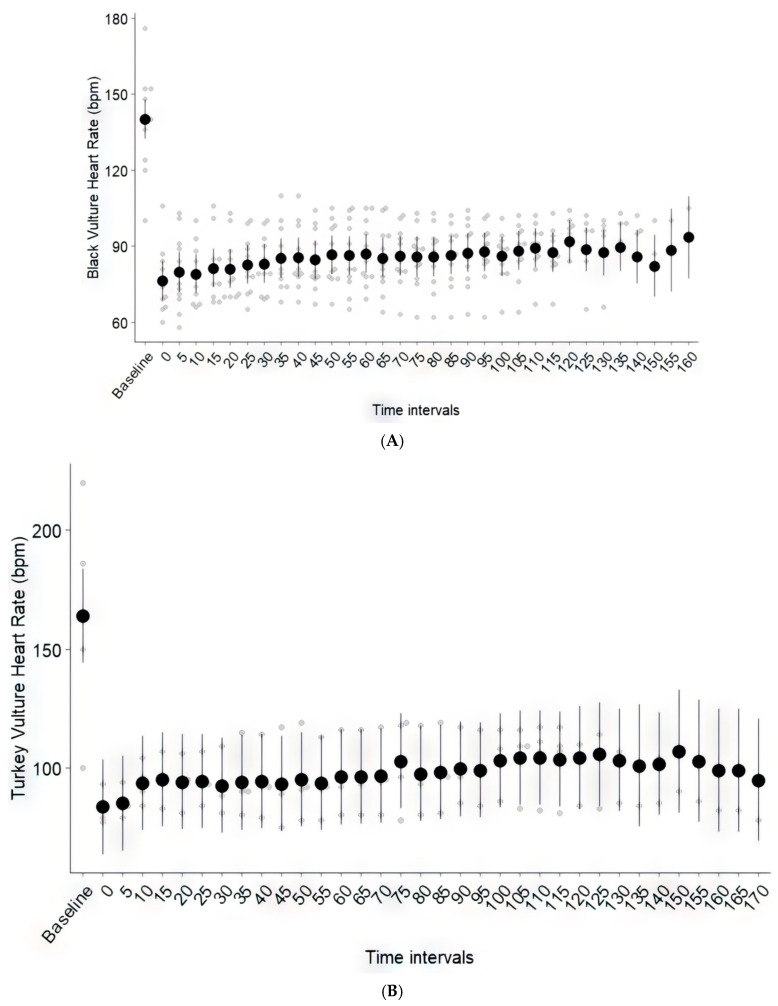
(**A**) Estimated marginal means of Black Vulture heart rate (beats per minute) across anesthesia time intervals, with standard error bars. Raw data points are shown in gray. All post-induction heart rate values (Time 0 onward) were significantly lower than the species’ awake baseline (*p* ≤ 0.001). (**B**) Estimated marginal means of Turkey Vulture heart rate (beats per minute) across anesthesia time intervals, with standard error bars. Raw data points are shown in gray. All post-induction heart rate values (Time 0 onward) were significantly lower than the species’ awake baseline (*p* ≤ 0.001).

Awake baseline HR and RR were measured in eleven Black Vultures and four Turkey Vultures, with no statistically significant differences observed between species (Table 1). Following DMBK administration, HR decreased significantly from baseline in both Black Vultures (F33,261 = 16.94, *p* < 0.001) and Turkey Vultures (F35,76 = 5.27, *p* < 0.001). This significant reduction was evident immediately at induction and persisted throughout the entire monitoring period (Figure 2A,B).

After induction, all the vultures were placed on pressure-controlled mechanical ventilation (10 breaths/min; 6 cmH_2_O PIP). This intervention also caused a statistically significant reduction in RR from baseline in both species, stabilizing at an estimated marginal mean of 7–13 breaths per minute in Black Vultures (F33,262 = 88.7, *p* < 0.001) and 8–12.5 breaths per minute in Turkey Vultures (F35,75 = 14.9, *p* < 0.001). No interspecies differences in RR were noted during ventilation, as both groups were maintained on identical ventilator settings, though this comparison was not statistically tested.

A detailed figure for RR overtime, analogous to Figure 2 for HR, is not presented. As all vultures were placed on pressure-controlled mechanical ventilation, RR was externally regulated and remained stable throughout the anesthetic period. This is consistent with the estimated marginal means for Black Vultures (7 to 13 breaths per minute) and Turkey Vultures (8 to 12.5 breaths per minute), as noted above, which were significantly lower than the awake baseline values.

The statistical results from the general linear mixed models are presented in Table 2, with the corresponding model-predicted values (Estimated Marginal Means) detailed in Table 3.

Analysis showed that oxygen saturation (SpO_2_, Supplemental Figure S2e) remained stable and consistent over time and between species, with values consistently maintained near 100%. Other variables demonstrated significant changes over time. End-tidal carbon dioxide, for instance, showed no species-specific difference but increased steadily in both groups throughout the procedure (Supplemental Figure S2f). Similarly, body temperature exhibited a significant decline over time in both species. While a visual trend suggested that Turkey Vulture temperatures dropped more rapidly (Supplemental Figure S2d), this interaction was not statistically significant.

Most notably, a highly significant Time × Species interaction was observed for all three blood pressure parameters: systolic, diastolic, and mean. This interaction indicates that cardiovascular trends diverged over time; pressures in Turkey Vultures tended to decrease, whereas pressures in Black Vultures tended to increase (Supplemental Figure S2a–c). The statistical significance of these effects from the linear mixed model is summarized in Table 3. The analysis of blood pressure also revealed more complex, species-specific responses. No significant differences were observed between groups for systolic or mean arterial pressure. However, while Turkey Vulture diastolic blood pressure eventually dropped below the level of Black Vultures, their overall mean diastolic blood pressure was significantly higher, as reflected in the estimated marginal mean values (Table 3). For the parameters analyzed with time as a categorical factor (Heart Rate and Respiratory Rate), Mean (SD) values at each point-in-time are provided for descriptive comparison in Supplementary Tables S5–S7.

Recovery from anesthesia was rapid and uneventful. Following the termination of isoflurane, all vultures quickly (within 1–3 min) resumed spontaneous breathing and were promptly extubated. The recovery quality was excellent, with all birds scoring 4 (Good) or 5 (Excellent) based on the standardized scoring system (Supplementary Table S4). The vultures regained normal alertness quickly; all were able to stand and maintain their balance within 5 min of extubation.

## 4. Discussion

This study found that DMBK premedication, followed by isoflurane maintenance, provides an effective anesthetic regimen for both Black and Turkey Vultures, characterized by stable cardiorespiratory parameters and smooth recoveries.

The 12–16 h fasting period used in this study appeared appropriate, as no regurgitation occurred during premedication, anesthesia, or recovery. Regurgitant observed during initial awake handling consisted primarily of liquid with minimal solid content, suggesting effective gastric emptying. Captive vultures in this study consumed 6 to 8 oz (approximately 170–225 g) of meat daily, provided ad libitum. Vultures of both species are capable of fasting for several days without deleterious effect [25,26]. Although gastric emptying times are not well documented in either species, their unique physiology, including a large crop and gorge-famine feeding behavior [3], may predispose them to perioperative regurgitation via drug-induced emesis or anesthetic-related esophageal relaxation. In this context, appropriate fasting and maintenance of anesthetic depth are critical to minimizing aspiration risk, especially given that endotracheal tube cuffs were intentionally left deflated.

Vultures are also known to regurgitate crop contents as a potent “fight or flight” mechanism [12,13,27]. In this study, 33% of the combined cohort exhibited such behavior during initial physical restraint. However, no regurgitation occurred after IM injection, indicating that the sedative and anxiolytic effects of the drug combination were sufficient to suppress this stress-induced response. The absence of regurgitation during the anesthetic period further supports the safety and efficacy of the protocol in managing both physiological and behavioral risks during immobilization.

A key finding was that the vultures in this study required notably lower drug dosages than those typically reported for other raptors. For instance, while published doses for dexmedetomidine in raptors range from 25 to 75 µg/kg [28], the vultures in this study were effectively sedated with only 5 µg/kg. Similarly, recommended ketamine doses often range from 10 to 20 mg/kg [18], whereas this study used 5 mg/kg. Midazolam is frequently used at 0.25–0.5 mg/kg [18], but a dose of 0.2 mg/kg was sufficient here. Finally, butorphanol is commonly cited at 1–6 mg/kg for analgesia in raptors [29], a range substantially higher than the 0.2 mg/kg used in this protocol. These reduced doses were selected conservatively to prioritize safety, given the limited species-specific data available for vultures. Rather than relying on a formal pilot study, we leveraged the known synergistic effects of a multimodal protocol and incorporated isoflurane mask induction as a contingency. Despite the low doses, the protocol provided safe and effective sedation. This outcome suggests that either the multimodal combination enhanced drug synergy, or vultures may be inherently more sensitive to these agents than other raptors, or that both factors contributed to the observed efficacy.

In this study, intravenous (IV) catheter placement and fluid administration were left out following risk-benefit analysis tailored to the species, procedure, and patient condition. All vultures were classified as ASA I (healthy) and confirmed to be well-hydrated prior to the 12–16 h fasting period. Given that ERG is a non-invasive procedure with no blood loss and minimal fluid loss, the potential risks of catheterization, including hematoma formation, vessel thrombosis, and infection, were deemed to outweigh the limited benefits of prophylactic fluid therapy. The procedure duration was approximately two hours, and all patients remained clinically stable throughout. Cardiovascular parameters, particularly BP, were consistently within normal limits during anesthesia, indicating no evidence of hypotension or compromise. The absence of adverse effects or hemodynamic instability suggests that omitting IV fluids and catheterization did not result in any clinically significant or catastrophic outcome and supports that this is an alternative approach for non-invasive procedures in healthy avian patients.

A critical component of this protocol was the management of the avian respiratory system, which differs markedly from that of mammals [18]. Unlike the elastic lungs of mammals, vultures possess rigid pulmonary structures ventilated by a series of air sacs [18,30,31]. Under anesthesia, spontaneous breathing in birds often leads to hypoventilation and variable elevations in arterial carbon dioxide, which can disrupt retinal perfusion and neuronal activity [32], compromising the precision required for ERG recordings. To eliminate this variability and maintain consistent physiological conditions across individuals, controlled mechanical ventilation was implemented. Pressure-controlled ventilation was specifically selected over volume-controlled modes due to its compatibility with the non-compliant nature of avian lungs, minimizing the risk of barotrauma [30,31,33]. In this study, a low PIP of approximately 6–8 cmH_2_O provided stable and effective ventilation throughout the procedure. Anesthetic depth was carefully tapered to suppress spontaneous respiratory efforts (“bucking”) during controlled ventilation, abolish nystagmus, and limit the corneal reflex, thereby ensuring stable respiratory support and complete immobilization for high-fidelity ERG acquisition.

Following airway establishment, selecting an appropriately sized endotracheal tube is a critical step in avian anesthesia. Unlike mammals, birds, such as vultures, possess complete, non-expandable tracheal rings, rendering them highly susceptible to pressure-related tracheal injuries [18,30,31,33,34]. Over-inflation of a standard cuffed endotracheal tube can result in mucosal necrosis, tracheal rupture, or stricture. To mitigate this risk, non-cuffed tubes are frequently recommended for avian species [18]. However, they often fail to achieve a complete seal, leading to anesthetic gas leakage and complicating the delivery of controlled ventilator pressures.

Before selecting a cuffed tube, we evaluated the use of a Cole tube, which features a tapered distal end designed to seal at the glottis [18]. This approach proved unsuitable for vultures requiring mechanical ventilation, as substantial leakage occurred immediately upon applying positive pressure. We therefore adopted a third strategy by placing a cuffed endotracheal tube with the cuff left deflated [18]. This method provided a secure airway with minimal leakage, likely due to the deflated cuff acting as a passive baffle that disrupted direct airflow escape through the glottis. Crucially, it also eliminated the risk of cuff-related tracheal injury, an important safety consideration during prolonged procedures.

One significant challenge in avian anesthesia involves adapting standard veterinary monitoring equipment, which was originally designed for mammals, to suit the distinct anatomical features of birds. In this study, we introduced several important modifications to establish a consistent monitoring protocol for vultures. Among these adjustments, cardiac rhythm monitoring produced a stable and interpretable ECG waveform using a Lead II configuration. This arrangement involved placing nontraumatic alligator clips on the wings to serve as the right and left arm leads, and on the left toe to represent the left leg lead. The resulting signal allowed for clear rhythm analysis with minimal interference, confirming its reliability for use in large raptors. The clinical value of this approach was demonstrated when it enabled immediate detection and continuous tracking of a second-degree atrioventricular (AV) block in one Black Vulture (Figure 1). This arrhythmia was identified as a Type I (Wenckebach) block, characterized by a progressive lengthening of the P-R interval until a QRS complex is dropped. This setup allowed us to monitor the arrhythmia’s stability throughout the procedure without requiring intervention.

Building on the adaptations developed for cardiac monitoring, one of the most significant challenges in avian anesthesia involves the limitations of pulse oximetry. Although the toe web proved to be a viable site for placing the oxygen saturation probe, standard pulse oximeters are calibrated for mammalian hemoglobin rather than avian. This discrepancy can lead to misleading readings, often underestimating oxygenation at higher saturation levels and overestimating it at lower levels [18,33,34,35]. These inaccuracies arise from physiological differences, as avian hemoglobin typically exhibits a higher affinity for oxygen compared to its mammalian counterpart. As a result, algorithms designed for human patients may misinterpret light absorption data, potentially concealing true hypoxemia behind a seemingly acceptable oxygen saturation value above 92 percent.

A similar issue occurs with arterial blood gas analysis. While partial pressure of oxygen (PaO_2_) can be measured directly, the calculated oxygen saturation remains unreliable due to the use of human-based algorithms in standard analyzers. Given these limitations and the impracticality of frequent arterial sampling, we chose to monitor oxygen saturation trends rather than rely on absolute values. Focusing on directional changes, particularly stable or rising patterns, proved to be a more practical and informative approach, even when the numerical values were physiologically skewed. This trend-based monitoring is clearly illustrated in the historical record shown in Figure 1.

To further support this assessment, we continuously monitored both FiO_2_ and EtO_2_. Observing consistently high values, typically above 85 percent, alongside a stable SpO_2_ trend provided strong indirect evidence that the vulture’s lungs were receiving oxygen-enriched gas, as shown in Figure 1. This combined approach, which interpreted oxygen saturation trends in the context of delivered oxygen concentrations, offered a practical and more reliable method for evaluating oxygenation status in anesthetized vultures.

Obtaining consistent non-invasive blood pressure (NIBP) readings in avian species is notoriously challenging [36,37]. However, we leveraged the large size of vultures to overcome this issue by placing a cuff, with a width approximately 40% of the limb’s circumference [36,37], on the leg shaft near the tibiotarsus joint. This approach yielded consistent SBP, DBP, and MBP measurements, enabling us to effectively monitor cardiovascular trends (Figure 1). These findings confirm that this specific technique and location are critical for assessing cardiovascular stability in vultures under anesthesia.

In addition to anatomical considerations, our protocol was tailored to address the distinctive physiological demands of avian patients. One key component was the use of side-stream capnography configured to a low-flow neonate setting (50 mL/min). This adjustment was essential, as standard aspiration rates designed for larger domestic animals can easily exceed a vulture’s tidal volume, resulting in sample dilution and inaccurate measurements of EtCO_2_ and Et-Iso. This issue is well-documented in the avian anesthesia literature, where high sampling rates have been shown to underestimate EtCO_2_ due to anatomical dead space and low respiratory volumes [36,38,39]. Despite these limitations, EtCO_2_ remains a valuable tool for monitoring ventilation trends and detecting hyperventilation or hypoventilation [36,38,39]. Additionally, placing the vultures on pressure-controlled ventilation likely helped compensate for any mismatch between delivered and measured ventilation.

Continuous monitoring of EtCO_2_ and Et-Iso trends provided consistent, real-time feedback on ventilatory status and anesthetic depth. This approach aligned with observed anesthetic requirements during ERG procedures, where the median Et-Iso concentration needed to maintain a stable plane of anesthesia was 1.4%. Although no published minimum anesthetic concentration (MAC) exists for Black or Turkey Vultures, the reported MAC for the Cinereous Vulture (Aegypius monachus) is 1.06% [40]. Our findings suggest that the actual MAC for the species studied may be higher than both the published value and the median concentration used. This is likely due to two factors: premedication with a multimodal DMBK combination, which has anesthetic-sparing effects, and the non-noxious nature of ERG procedures, which require immobilization rather than deep analgesia. Accordingly, the Et-Iso concentration used here would be expected to fall below the true MAC, which is defined by response to a noxious stimulus.

A significant reduction in HR from the awake baseline was observed in both species following DMBK premedication and isoflurane anesthesia. This bradycardia is a well-documented and expected effect of dexmedetomidine, a potent alpha-2 adrenergic agonist [18,31,33]. Dexmedetomidine slows HR through two primary mechanisms: a centrally mediated sympatholytic action that reduces sympathetic tone, and a peripherally mediated vasoconstriction that activates a baroreceptor reflex, further suppressing HR. In this combination, the potent bradycardic effects of dexmedetomidine clearly superseded the known tachycardic properties of ketamine. Critically, this bradycardia was not associated with hypotension or other adverse events, suggesting it represented a stable, compensatory response rather than a sign of cardiovascular decompensation.

A related and notable finding was the maintenance of mean blood pressure (MBP) at levels considered hypertensive in commonly anesthetized domestic species such as dogs and cats [18,31,32]. This elevation is a direct consequence of the profound peripheral vasoconstriction induced by dexmedetomidine, likely compounded by the sympathomimetic effects of ketamine. While a transient hypertensive phase is typical in mammals, the sustained nature of this elevation, even during isoflurane maintenance, may reflect a species-specific cardiovascular response in vultures. Unlike the hypotensive trends often observed in anesthetized small mammals, this profile aligns more closely with findings in other avian species. For instance, elevated MBP has also been reported in raptors such as red-tailed hawks (Buteo jamaicensis) under volatile anesthesia. Zehnder et al. [41] noted similarly high values during indirect blood pressure monitoring in anesthetized red-tailed hawks, suggesting that elevated pressures may be a common feature among raptors rather than a protocol-specific outcome. These observations highlight the importance of intra-avian comparisons over extrapolation from mammalian norms when interpreting cardiovascular responses under anesthesia.

A statistically significant difference in diastolic blood pressure (DBP) was observed between species (*p* = 0.033), with Turkey Vultures showing a higher mean DBP. Although this may reflect a genuine species-specific variation, such as a more pronounced vasoconstrictive response to dexmedetomidine or differing sensitivity to isoflurane-induced vasodilation, the small sample size for Turkey Vultures (*n* = 4) warrants cautious interpretation. Clinically, the approximate 13 mmHg difference may be of limited significance, as DBP remained markedly elevated in both species (126.18 mmHg in Black Vultures and 139.16 mmHg in Turkey Vultures).

Overall, the cardiovascular profile observed in these vultures, characterized by bradycardia and sustained hypertension, appears well tolerated and may represent a normal anesthetic plane for these avian species. This reinforces the need for species-specific baseline data and cautions against relying on mammalian norms when managing anesthesia in avian species.

The multimodal anesthetic agents used in this study, including dexmedetomidine, midazolam, and butorphanol, each have specific antagonists: atipamezole, flumazenil, and naloxone, respectively. These reversal agents can play a crucial role when a shortened recovery time is desired. We chose not to use them in this study because the anesthetic doses were low and followed by two hours of isoflurane maintenance, which likely allowed sufficient drug metabolism. This was supported by the vultures’ consistently smooth and rapid recoveries. Nevertheless, these antagonists remain valuable clinical tools if a more expedited recovery is needed or an emergency arises.

It is important to acknowledge several limitations of this study. The primary limitation is the small sample size, particularly for Turkey Vultures, which restricts the statistical power of interspecies comparisons and may not capture the full range of individual physiological responses. Furthermore, while the monitoring techniques were effective for clinical trend analysis, their absolute accuracy has known constraints. Specifically, the NIBP values were not validated against the gold standard of direct arterial pressure, and the pulse oximetry readings are subject to the inherent inaccuracies of devices calibrated for human hemoglobin. Additionally, the absence of IV catheterization, while justified by the stable cardiovascular performance of the vultures, meant that immediate IV access was unavailable in the unlikely event of an adverse anesthetic reaction. Respiratory function was also controlled throughout the procedures to maintain a consistent and appropriate range of arterial partial pressure of CO_2_ (PaCO_2_), which was necessary for reliable ERG recordings. As a result, any anesthetic protocol-induced respiratory depression could not be properly evaluated under these conditions. Finally, the scope of the study was focused, as the anesthetic protocol was evaluated only during a non-painful procedure in healthy adult vultures. Consequently, its efficacy for more invasive surgeries, compromised patients, or other raptor species remains to be determined.

## 5. Conclusions

In conclusion, this study demonstrates that a multimodal anesthetic protocol, combined with practical adaptations of standard mammalian monitoring techniques, is both safe and effective for Black and Turkey Vultures. The findings establish a foundational approach to anesthetizing these species, offering a repeatable regimen supported by detailed monitoring standards. This protocol may serve as a useful template for improving anesthetic management and procedural safety in other similarly sized raptors.

## Figures and Tables

**Table 1:** Comparison of Body Weight and Anesthetic Parameters Between Black Vultures (*n* = 11) and Turkey Vultures (*n* = 4).

Parameter	Black Vulture	Turkey Vulture	*p*-Value (Between Species)
Body Weight (kg) ^1^	1.88 ± 0.30	1.93 ± 0.10	0.689
Induction Score ^2^	3 (1–5)	4 (3–4)	0.223
Endotracheal Tube Size (mm) ^1^	3.09 ± 0.30	3.00 ± 0.00	0.341
Total Anesthesia Time (min) ^1^	138.27 ± 17.54	139.25 ± 14.34	0.916
ERG Recording Time (min) ^1^	109.00 ± 14.39	116.25 ± 11.12	0.338
Recovery Score ^2^	4 (4–5)	5 (4–5)	0.101
Awake Heart rate ^1^	138.8 ± 20.8	164.0 ± 51.35	0.404
Awake Respiratory Rate ^1^	48.6 ± 11.0	52.5 ± 18.7	0.716

^1^ Values are presented as mean ± standard deviation. Group comparisons were performed using a two-sample *t*-test. ^2^ Values are presented as median (range). Group comparisons were performed using the Mann–Whitney U test.

**Table 2:** Effects of Time and Species on Cardiorespiratory and Temperature Variables: Results from General Linear Mixed Model Analysis.

Factor	Degrees of Freedom	F-Statistic	* *p*-Value
**Oxygen Saturation (SpO_2_)**			
Time elapsed (min)	1, 387	0.32	0.573
Species	1, 14	0.48	0.498
Time:Species	1, 387	1.14	0.287
**End-tidal CO_2_ (EtCO_2_)**			
Time elapsed (min)	1, 389	14.69	**<0.001**
Species	1, 14	0.68	0.424
Time:Species	1, 389	2.45	0.119
**End-tidal Isoflurane (Et-Iso)**			
Time elapsed (min)	1, 381	4.21	**<0.001**
Species	1, 13	2.28	0.131
Time:Species	1, 381	0.04	0.850
**Systolic Blood Pressure**			
Time elapsed (min)	1, 383	26.46	**<0.001**
Species	1, 13	2.79	0.118
Time:Species	1, 383	114.51	**<0.001**
**Diastolic Blood Pressure**			
Time elapsed (min)	1, 388	10.26	**<0.001**
Species	1, 14	5.59	**0.033**
Time:Species	1, 388	110.44	**<0.001**
**Mean Arterial Pressure**			
Time elapsed (min)	1, 387	13.47	**<0.001**
Species	1, 14	4.19	0.060
Time:Species	1, 387	97.86	**<0.001**
**Temperature (˚F)**			
Time elapsed (min)	1, 389	14.69	**<0.001**
Species	1, 14	0.68	0.424
Time:Species	1, 389	2.45	0.119

* Bolded *p*-values indicate statistical significance.

**Table 3:** Overall Estimated Marginal Mean (EMM) ± Standard Error for Cardiorespiratory and Temperature Variables in Anesthetized Vultures. * Indicates a significant difference between species (*p* = 0.033).

Variable	Black Vulture (*n* = 11)	Turkey Vulture (*n* = 4)
**SpO_2_ (%)**	99.15 ± 0.39	99.53 ± 0.65
**Systolic BP (mmHg)**	183.24 ± 9.60	187.46 ± 15.92
Diastolic BP (mmHg) *	126.18 ± 8.63	139.16 ± 14.31
**Mean Arterial BP (mmHg)**	149.01 ± 8.96	157.53 ± 14.85
**EtCO_2_ (mmHg)**	43.05 ± 1.57	39.55 ± 2.60
**Et-Iso (%)**	1.25 ± 0.05	1.39 ± 0.08
**Temperature (°F)**	99.52 ± 0.44	99.19 ± 0.66

## Data Availability

The original contributions presented in this study are included in the article/Supplementary Material. Further inquiries can be directed to the corresponding author.

## Supplementary Materials

The following supporting information can be downloaded at: https://www.mdpi.com/article/10.3390/vetsci12111091/s1. Table S1: Sedative Transition Quality Score: Evaluation of the transition from wakefulness to deep sedation, rated on a scale from 1 (rough) to 5 (excellent). Table S2: Final Sedative Degree Score (Vulture Sedation Scale): Assessment of posture and responsiveness at the 15 min mark, rated on a scale from 1 (Alert) to 5 (Deep Sedation- Unarousable). Refer to Figure S1 for the vulture ethogram illustrating sedation behaviors. Table S3: Anesthetic Induction Quality Score: Evaluation of the transition into general anesthesia using isoflurane via face mask, rated on a scale from 1 (poor) to 5 (good). Table S4: Anesthesia Recovery Score: Assessment of recovery quality based on vulture behavior from extubation to achieving an alert, standing posture, rated on a standardized 5-point scale from 1 (poor) to 5 (excellent). Table S5: Arithmetic means ± standard deviations and sample size of Black Vulture and Turkey Vulture heart rates at each time interval under anesthesia. Table S6: Arithmetic means ± standard deviations and sample size of Black Vulture and Turkey Vulture respiration rates at each time interval under anesthesia. Table S7: Arithmetic means and standard deviations of cardiorespiratory parameters measured across the anesthetic process in Black Vultures and Turkey Vultures. Note that these means include multiple measurements from the same individuals, and data are not independent (for the proper estimation, see marginal means in the main text). Figure S1: Final Sedative Degree Score in Vultures. This ethogram, a catalog of an animal’s behaviors, was created to document the sedative states of vultures. This figure represents a visual reference for the sedation scores observed in Black and Turkey Vultures. Numerical values correspond to the assessed depth of sedation (see Table S2 for Final Sedation Score criteria), while the accompanying illustrations depict the characteristic posture and physical appearance associated with each score. This graph was generated by converting realistic photos into AI-rendered figures for clarity. For details on the process, please refer to the Acknowledgments section. Figure S2: Interaction plots of physiological trends in Black Vultures and Turkey Vultures under general anesthesia. Plots show interactions of time with (a) Systolic Blood Pressure (SBP), (b) Diastolic Blood Pressure (DBP), (c) Mean Arterial Pressure (MBP), (d) Body Temperature, (e) Oxygen Saturation (SpO2), and (f) End-tidal Carbon Dioxide (EtCO_2_) over the anesthesia time interval. Trends for Black Vultures are represented by the solid blue line, and trends for Turkey Vultures are represented by the dashed orange line. Shaded areas represent the 95% confidence intervals.
