# Supplementary material for: Evaluation of a Multimodal Anesthetic Protocol for Immobilization in Black Vultures (Coragyps atratus) and Turkey Vultures (Cathartes aura)
Source: Vet Sci. 2025 Nov 16;12(11):1091. doi: 10.3390/vetsci12111091 (PMC12656902; doi:10.3390/vetsci12111091)
Supplement: Supplementary file 1 [file vetsci-12-01091-s001.zip › Supplemental Tables and Figure.pdf]

Supplemental Tables S1–S4, Figures S1 and S2

Supplementary Materials: The following supporting information is available for download: Appendix: Vulture Sedative Transition Quality Score, Anesthetic Scoring Rubrics.

Table S1: Sedative Transition Quality Score: Evaluation of the transition from wakefulness to deep sedation, rated on a scale from 1 (rough) to 5 (excellent).

Score	Quality	Description
**1**	Rough	Exhibits extreme and uncontrolled excitatory movements, such as repeated stumbling, violent wing-flapping, somersaulting, and continuous or frequent rolling. Retching or active vomiting is characteristic.
**2**	Poor	Shows similar excitatory behaviors that are less frequent or violent, with intermittent periods of quietness. Vomiting or retching may still be present.
**3**	Acceptable	Becomes sedate with only occasional stumbling and attempts to maintain sternal recumbency, sometimes in an awkward posture. No vomiting or retching is observed.
**4**	Good	A smooth transition to moderate or deep sedation with only minimal, infrequent attempts to adjust body position. No vomiting or retching.
**5**	Excellent	A rapid, smooth, and quiet transition to a profound level of sedation, calmly assuming a stable, sternal recumbency without arousal or excitation.

Table S2: Final Sedative Degree Score (Vulture Sedation Scale): Assessment of posture and responsiveness at the 15-minute mark, rated on a scale from 1 (Alert) to 5 (Deep Sedation- Unarousable). Refer to Figure A1 for the vulture ethogram illustrating sedation behaviors.

Scale	Degree	Description
**1**	Alert	The vulture stands upright with its head raised, remaining fully responsive and coordinated.
**2**	Mild Sedation	The head lowers slightly, eyes are semi-closed, and wings begin to droop.
**3**	Moderate Sedation	Wings are loose and down, the head is drooping with eyes closed, but the vulture remains standing.
**4**	Profound Sedation	Cannot support its own body weight; wings hang loosely, head and neck are flaccid, and tarsometatarsi are touching the ground.
**5**	Deep Sedation (Unarousable)	The body is fully relaxed and unresponsive, with no movement or reaction to external stimuli.

Table S3: Anesthetic Induction Quality Score: Evaluation of the transition into general anesthesia using isoflurane via face mask, rated on a scale from 1 (poor) to 5 (good).

Score	Quality	Description
**1**	Poor	Vulture is awake or lightly sedated, exhibiting continuous and profound struggling with its legs and wings. It actively fights the face mask, and its beak cannot be opened without significant force. Intubation is difficult and prolonged. Regurgitation occurs during the struggle or mask induction.
**2**	Fair	Moderate struggling occurs during handling or application of the face mask. The vulture resists opening its beak, requiring some effort to achieve intubation. The transition to a stable anesthetic plane is delayed but eventually successful.
**3**	Acceptable	The vulture is calm but shows minor resistance, such as head movement or brief tensing of the body. The beak can be opened with minimal effort. Intubation is straightforward but not immediate.
**4**	Good	The vulture is deeply sedate and offers no resistance. The beak opens easily, and the face mask is well-accepted. A clear swallowing reflex is still present, requiring a brief period of face mask isoflurane (1-2 minutes) for a smooth intubation.
**5**	Excellent	The vulture is profoundly sedate with sufficient muscle relaxation. The beak opens with no resistance, and while intubation is possible, the vulture does not fully tolerate the insertion of the endotracheal tube due to a minimal gag or swallow reflex. A very brief application of face mask isoflurane (<1 minute) is all that is needed to achieve a rapid and seamless intubation.

Table S4: Anesthesia Recovery Score: Assessment of recovery quality based on vulture behavior from extubation to achieving an alert, standing posture, rated on a standardized 5-point scale from 1 (poor) to 5 (excellent).

Score	Quality	Description
**1**	Poor	A prolonged and violent recovery characterized by frequent, intense struggling and rolling. After the excitatory phase, the vulture remains deeply sedated, unable to be aroused, and can still be handled easily due to the significant sedative "hangover." The bird is unable to stand for an extended period.
**2**	Fair	Significant struggling is present, but it is less frequent or less intense than a score of 1. The bird has considerable difficulty gaining body control and remains in a sedated, unstable posture for a prolonged time (approximately 30 minutes) before stabilizing.
**3**	Acceptable	The vulture is able to stand briefly but repeatedly falls back into a sedated posture (head drooping, wings loose, tarsometatarsi on the floor). This period of relapse lasts for a noticeable duration (10-15 minutes) before full alertness is achieved.
**4**	Good	A rapid recovery where the vulture quickly regains body control and stands with its eyes open. It may show occasional, brief signs of relapse, such as slight head dropping or minor imbalance, but is easily aroused and quickly self-corrects.
**5**	Excellent	A rapid, smooth, and calm recovery. The vulture regains full body control and alertness quickly, stands steadily, and shows no signs of struggle, excitation, or relapses into sedation.

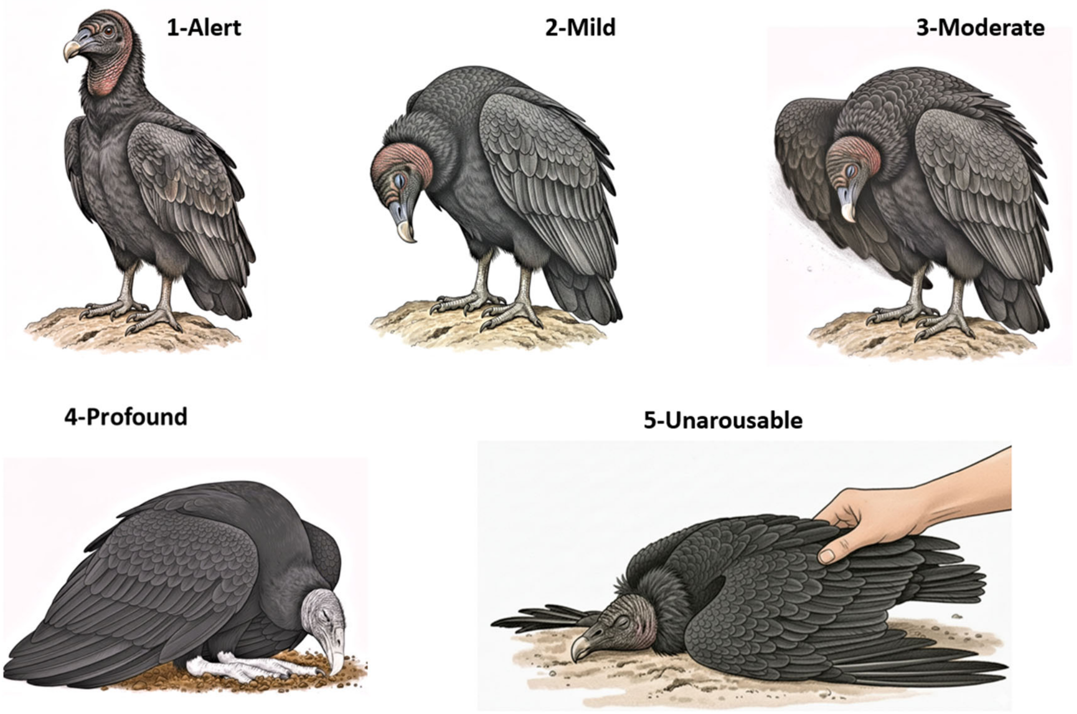

Figure S1: Final Sedative Degree Score in Vultures. This **ethogram,** a catalog of an animal's behaviors, was created to document the sedative states of vultures. This figure represents a visual reference for the sedation scores observed in Black and Turkey Vultures. Numerical values correspond to the assessed depth of sedation (see Table A2 for Final Sedation Score criteria), while the accompanying illustrations depict the characteristic posture and physical appearance associated with each score. This graph was generated by converting realistic photos into AI-rendered figures for clarity. For details on the process, please refer to the Acknowledgments section.

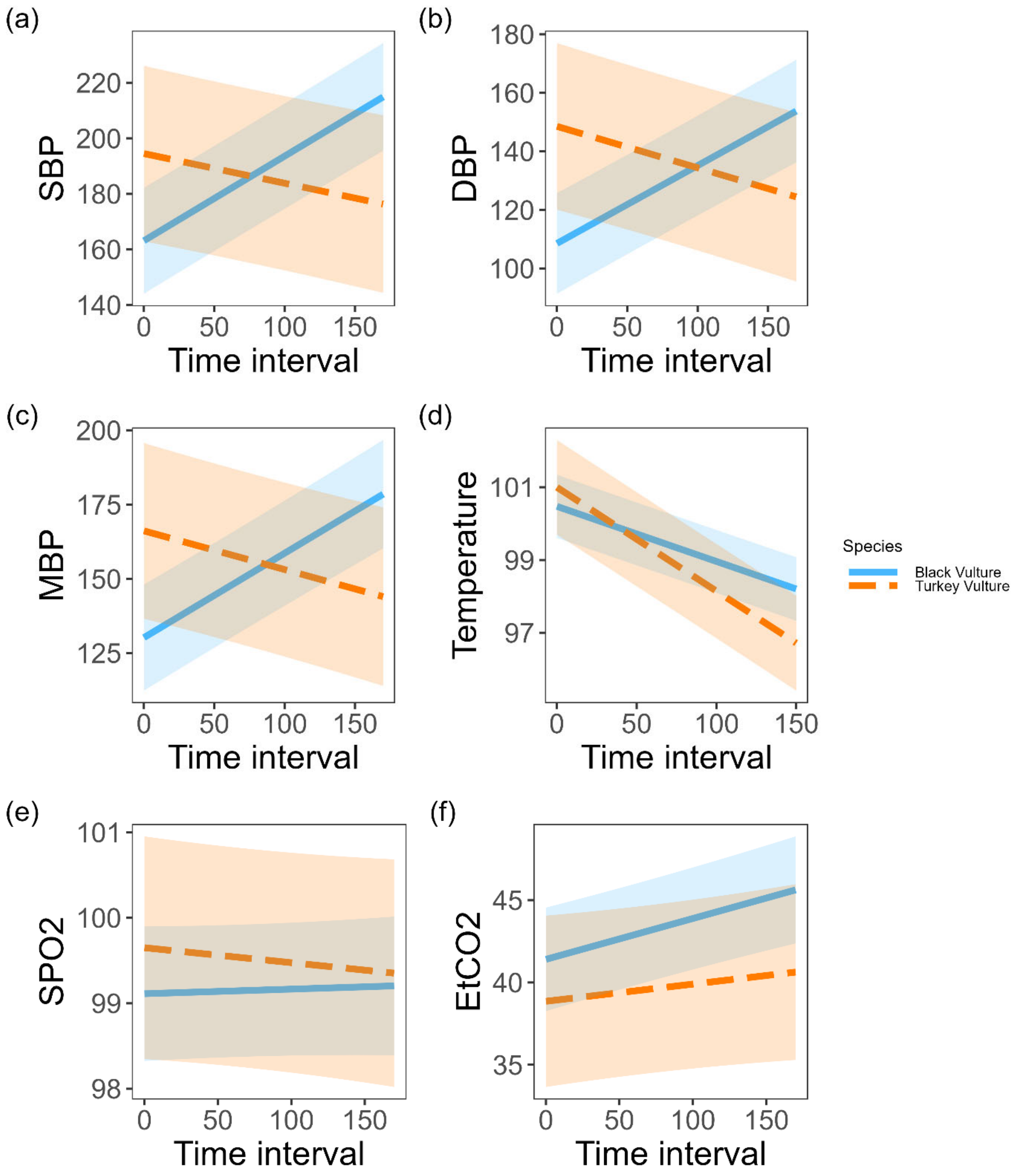

Supplemental Figure S2. Interaction plots of physiological trends in Black Vultures and Turkey Vultures under general anesthesia. Plots show interactions of time with (a) Systolic Blood Pressure (SBP), (b) Diastolic Blood Pressure (DBP), (c) Mean Arterial Pressure (MBP), (d) Body Temperature, (e) Oxygen Saturation (SpO2), and (f) End-tidal Carbon Dioxide (EtCO2) over the anesthesia time interval. Trends for Black Vultures are represented by the solid blue line, and trends for Turkey Vultures are represented by the dashed orange line. Shaded areas represent the 95% confidence intervals.
