# Supplementary material for: Evaluation of a Multimodal Anesthetic Protocol for Immobilization in Black Vultures (Coragyps atratus) and Turkey Vultures (Cathartes aura)
Source: Vet Sci. 2025 Nov 16;12(11):1091. doi: 10.3390/vetsci12111091 (PMC12656902; doi:10.3390/vetsci12111091)
Supplement: Supplementary file 1 [file vetsci-12-01091-s001.zip › Supplementary Arithmetic Mean Table 5A-C.pdf]

Supplementary Table S5

Arithmetic means ± standard deviations and sample size of Black Vulture and Turkey Vulture heart rates at each time interval under anesthesia.

Time	Black Vulture Mean Heart Rate (bpm)	Black Vulture Sample Size	Turkey Vulture Mean Heart Rate (bpm)	Turkey Vulture Sample Size
Baseline	138.8±20.8	11	164.0±51.4	4
0	76.3±12.9	11	83.5±7.2	4
5	79.7±15.0	11	85.0±6.4	4
10	78.9±11.4	11	93.5±8.5	4
15	81.3±12.6	11	95.0±9.9	4
20	80.9±11.9	11	94.0±10.2	4
25	82.6±11.4	11	94.3±9.5	4
30	83.0±11.3	11	92.5±11.9	4
35	85.2±12.9	11	93.8±14.9	4
40	85.5±12.6	11	94.3±14.5	4
45	84.5±11.8	11	93.3±17.5	4
50	86.5±12.3	11	95.0±17.2	4
55	86.2±14.1	11	93.5±14.5	4
60	87.0±12.7	11	96.0±15.0	4
65	85.3±13.2	11	96.3±14.9	4
70	86.0±11.7	11	96.5±15.3	4
75	85.7±11.7	11	102.8±19.6	4
80	85.6±12.3	11	97.5±15.8	4
85	86.5±11.8	11	98.0±15.7	4
90	87.3±12.6	11	99.5±13.3	4
95	87.7±11.3	11	99.0±13.1	4
100	86.0±9.7	11	103.0±12.7	4
105	88.2±10.8	11	104.3±14.5	4
110	89.3±9.6	11	104.3±15.4	4
115	87.3±9.8	10	103.5±15.6	4
120	95.4±6.6	7	97.0±18.4	2
125	90.3±13.8	6	98.5±21.9	2
130	90.8±14.0	5	96.0±15.6	2
135	98.3±4.6	4	84.0	1
140	97.7±3.8	3	94.5±13.4	2
145	95.3±5.7	3	84.0	1
150	93.5±9.2	2	90.0	1
155	100.0	1	86.0	1
160	105.0	1	82.0	1
165	NA	NA	82.0	1
170	NA	NA	78.0	1

Supplementary Table S6

Arithmetic means ± standard deviations and sample size of Black Vulture and Turkey Vulture respiration rates at each time interval under anesthesia.

Time	Black Vulture Mean Respiration Rate	Black Vulture Sample Size	Turkey Vulture Mean Respiration Rate	Turkey Vulture Sample Size
Baseline	48.6±11.0	11	52.5±18.6	4
0	9.4±1.6	11	10.0±0.0	4
5	9.8±0.6	11	10.0±0.0	4
10	9.8±0.6	11	10.0±0.0	4
15	9.7±0.6	11	10.0±0.0	4
20	9.7±0.6	11	10.0±0.0	4
25	9.7±0.6	11	10.0±0.0	4
30	9.7±0.6	11	10.0±0.0	4
35	9.8±0.4	11	10.0±0.0	4
40	9.9±0.3	11	10.0±0.0	4
45	9.8±0.4	11	10.0±0.0	4
50	9.9±0.3	11	10.0±0.0	4
55	10.1±0.7	11	10.0±0.0	4
60	10.1±0.7	11	10.0±0.0	4
65	10.1±0.7	11	10.0±0.0	4
70	10.1±0.7	11	10.0±0.0	4
75	10.1±0.7	11	10.0±0.0	4
80	10.1±0.7	11	10.0±0.0	4
85	10.1±0.7	11	10.0±0.0	4
90	10.1±0.7	11	10.0±0.0	4
95	10.1±0.7	11	10.0±0.0	4
100	10.1±0.7	11	10.0±0.0	4
105	9.3±2.8	11	10.0±0.0	4
110	10.1±0.7	11	10.0±0.0	4
115	10.2±0.6	10	10.0±0.0	4
120	10.6±1.0	7	10.0±0.0	2
125	10.7±1.0	6	10.0±0.0	2
130	10.0±2.4	5	10.0±0.0	2
135	13.8±4.3	4	10.0	1
140	11.3±1.2	3	12.5±3.5	2
145	7.7±2.1	3	10.0	1
150	9.5±0.7	2	10.0	1
155	10.0	1	10.0	1
160	10.0	1	12.0	1
165	NA	NA	11.0	1
170	NA	NA	8.0	1

Supplementary Table S7: Arithmetic means and standard deviations of cardiorespiratory parameters measured across the anesthetic process in Black Vultures and Turkey Vultures. Note that these means include multiple measurements from the same individuals, and data are not independent (for the proper estimation, see marginal means in the main text).

**Parameter**	**Black Vulture Arithmetic Mean ± SD**	**Turkey Vulture Arithmetic Mean ± SD**
Temperature (˚F)	99.5 ± 1.3	99.2 ± 1.8
SpO_2	99.2 ±1.5	99.5 ±1.0
Systolic Blood Pressure	180.9 ± 35.4	187.8 ± 25.9
Diastolic Blood Pressure	123.7 ± 33.2	138.7 ± 19.0
Mean Arterial Pressure	146.5 ± 33.0	157.0 ±27.1
$EtCO_2$	43.4 ± 7.1	39.5 ± 3.7
